# Individual differences in the use of the response scale determine valuations of hypothetical health states: an empirical study

**DOI:** 10.1186/1472-6963-7-62

**Published:** 2007-04-27

**Authors:** Marie-Louise Essink-Bot, Marja C Stuifbergen, Willem-Jan Meerding, Caspar WN Looman, Gouke J Bonsel, the VOTE group

**Affiliations:** 1 Department of Public Health, Erasmus MC/University Medical Center Rotterdam, PO Box 2040, 3000 CA Rotterdam, The Netherlands; 2 Department of Social Medicine – Public Health Methods, Amsterdam Medical Center, Amsterdam, The Netherlands; 3 Present address: Julius Center for Health Sciences and Primary Care, UMC Utrecht, Utrecht, The Netherlands; 4 Present address: Pfizer bv, Capelle a/d IJssel, The Netherlands; 5 Present address: Department of Health Policy and Management, Erasmus MC/University Medical Center Rotterdam, The Netherlands

## Abstract

**Background:**

The effects of socio-demographic characteristics of the respondent, including age, on valuation scores of hypothetical health states remain inconclusive. Therefore, we analyzed data from a study designed to discriminate between the effects of respondents' age and time preference on valuations of health states to gain insight in the contribution of individual response patterns to the variance in valuation scores.

**Methods:**

A total of 212 respondents from three age groups valued the same six hypothetical health states using three different methods: a Visual Analogue Scale (VAS) and two variants of the Time trade-off (TTO). Analyses included a generalizability study, principal components analysis, and cluster analysis.

**Results:**

Valuation scores differed significantly, but not systematically, between valuation methods. A total of 36.8% of variance was explained by health states, 1.6% by the elicitation method, and 0.2% by age group. Individual differences in the use of the response scales (e.g. a tendency to give either high or low TTO scores, or a high or low scoring tendency on the VAS) were the main source of remaining variance. These response patterns were not related to age or other identifiable respondent characteristics.

**Conclusion:**

Individual response patterns in this study were more important determinants of TTO or VAS valuations of health states than age or other respondent characteristics measured. Further valuation research should focus on explaining individual response patterns as a possible key to understanding the determinants of health state valuations.

## Background

The time trade-off (TTO) method is commonly used to elicit values for health states that can subsequently be used in the calculation of quality adjusted life-years (QALYs). It was designed by Torrance [1] as a less complicated, conceptually different but equally valid alternative to the standard gamble (SG) technique. In a TTO task, respondents express their preference for a given suboptimal health state by considering the number of life-years they are hypothetically willing to sacrifice in order to attain perfect health. Thus, the valuation task includes a hypothetical trade-off between length and quality of life. Trade-off techniques for valuations of health states are considered to comply more with the premises of utility theory than direct ratings of health states using visual analogue scaling [2].

Whether systematic variation is present in preferences of health states across population subgroups differing by socio-demographic characteristics, especially age, remains unresolved. For example, Sackett & Torrance [3] found an age effect for some but not all health state valuations with the TTO. Dolan et al. found an age effect on TTO values in a large group, but the variance explained by age was only 1–3% [4]. In contrast, Carter et al. [5] and Rosser & Kind [6] found no evidence that health state valuations correlated with age, sex or socioeconomic status. Recently, Wittenberg showed that SG or TTO preferences for health states varied by age of the respondent: valuations decreased 0.02 on a scale of 0–1 for every decade increase in age [7]. In contrast, others found that older subjects provided higher preference scores [8,9]. The presence of an effect of respondent characteristics could not be demonstrated in recent studies [10,11], whereas Froberg & Kane [12] attributed the absence of correlations between respondent characteristics and health state valuations to small sample sizes and a large variability in preference scores. Thus, there are mixed reports regarding the effect of respondent age on health state preferences and the main source of variability in these scores remains inconclusive [13].

Therefore, we designed a study to discriminate between the effects of respondents' age and time preference on valuations for hypothetical health states. We analyzed whether individual response patterns, defined as systematic differences among respondents in their use of the response scales, provided an alternative explanation for the variance in the valuation scores.

## Methods

### Rationale of the study design

Trade-off techniques for valuing health states assess how much of something valuable a respondent is hypothetically prepared to give up to improve health or to prevent getting worse. With the SG, the commodity to trade is the probability of surviving; with the willingness-to-pay, it is money; and with the TTO, life expectancy. However, the trading that is inherent in these methods is complex, because preferences elicited by these techniques incorporate both the preferences for health states and preferences for the commodity that is hypothetically being traded in exchange for better health [14]. Thus, preferences elicited by SG are to some extent affected by risk attitude, and preferences elicited by TTO by a respondent's time preference. For QALYs calculated from TTO-based preferences this may implicitly lead to double discounting [15]. Robinson et al. suggested a threshold of tolerance to describe the reluctance to trade any amount of life expectancy for a variety of relatively good health states that is often observed in TTO studies [16]. Such a threshold may arise if respondents value life years to come as being too valuable to sacrifice, even in a hypothetical situation, for a relatively small change from almost good health to optimal health [17]. An empirical comparison of two variants of the TTO method suggested that time preference partly explained the response variations among respondents [17].

If a respondent's age is a determinant of preferences for health states elicited with TTO, then two alternative explanations are theoretically available. Systematic variations in TTO results between older and younger respondents may arise if older respondents hold different preferences for health or for longevity. If respondents of different ages do hold different values for health states, this may be due either to a differing preference structure by age towards health per se, or to a correlation of respondent age with other characteristics that affect health state valuations, e.g. worse health [18,19]. If respondents of different ages have a different time preference, then responses to TTO scores will be higher among older respondents even if preferences for health states are not different across age groups.

In the present study, respondents from three age groups valued a set of six health states using a visual analogue scale (VAS) and two variants of the TTO: one standard TTO, anticipating 10 years in ill health (TTO-10), and one involving a lifetime perspective, anticipating ill health during the remaining lifetime of each respondent, equal to each individual's actuarial life expectancy (TTO-LE). We hypothesized that VAS scores would not be affected by time preference. In TTO-LE, older respondents were presented with a lower total number of years remaining for hypothetical trading than younger respondents, so that an effect of differences in time preference on the elicited valuations would be most prominent in this TTO variant [20]. In TTO-10, confronting respondents from all age groups with the same hypothetical life expectancy of 10 years, effects of different preferences for health and time were expected to be mixed.

### Ethics approval

The Medical Ethics Review Board of Erasmus MC – University Medical Center Rotterdam, The Netherlands, approved the study (MEC 195.795/2000/208; November 14, 2000). All subjects who participated provided written informed consent.

### Respondents

Respondents were recruited from the general population in the Rotterdam area in 2003. We aimed to recruit from 3 age groups representing different stages in life: i.e., approximately 20–25 (age group 1), 45–50 (age group 2), 60–65 (age group 3) years, respectively. Respondents could choose to complete the computerized valuation questionnaire at home, or at the university in groups of about 20 respondents. A research assistant was present, who had instruction to influence the content of the participants' responses to the questionnaire in no way. The participants completed the questionnaires individually and had adequate privacy.

### Health states

Six hypothetical health state descriptions were constructed using the modified EQ-5D+ classification system, as developed by Krabbe et al. [21] (Table 1).

**Table 1:** EuroQol 5D+ classification of health

Dimension	Level	Code
Mobility	No problems in walking about	1
	Some problems in walking about	2
	Confined to bed	3
Self-care	No problems with washing or dressing self	1
	Some problems with washing and dressing self	2
	Unable to wash or dress self	3
Usual activities	No problems with performing usual activities (study, housework, family or leisure activities)	1
	Some problems with performing usual activities	2
	Unable to perform daily activities	3
Pain/discomfort	No pain or discomfort	1
	Moderate pain or discomfort	2
	Extreme pain or discomfort	3
Anxiety/depression	Not anxious or depressed	1
	Moderately anxious or depressed	2
	Extremely anxious or depressed	3
Cognition	No problems in cognitive functioning (e.g. memory, concentration, coherence, IQ)	1
	Some problems in cognitive functioning	2
	Extreme problems in cognitive functioning	3

The health states were chosen to represent a broad spectrum of severity and affected attributes. The health states were presented in the questionnaire in the following order: 222111, 112121, 222223, 122122, 332221 and 123121. The order of presentation was similar across valuation methods. The figures in the description correspond to the EQ-5D+ system. The first figure indicates the level (no, some, or severe problems) on the first attribute (mobility), the second the level on the second attribute (self-care), and so on. Thus, health state 222111 represents a health state with some problems in walking about, some problems with washing and dressing oneself, some problems with performing usual activities, no pain or discomfort, no anxiety or depression, and no problems in cognitive functioning.

All respondents valued all six health states by all three methods in the same order.

### Questionnaire

The valuation tasks were included in an interactive internet-enabled self-report valuation questionnaire through a generic (adaptable) Internet-tool, using PHP (version 4.0.1 and higher), MySQL (version 3.22 and higher), and JavaScript (version 1.3) [22]. The questionnaire consisted of the following elements in the sequence presented below:

- Ranking of six health states: as a prelude to the actual valuation tasks, respondents were asked to rank the six health states in order of severity.

- Visual analogue scale (VAS): The six health states were presented in the ranking order the respondent had chosen and were valued on a vertical VAS with labeled endpoints "death" and "full health" for values 0 and 100, respectively.

- Time trade-off with life expectancy (TTO-LE) of the 6 health states. For each health state separately, respondents had to choose between living their actual remaining life expectancy in health state x, or to live a shorter period of time in full health. For each respondent, actual life expectancy was estimated based on the respondent's age and tables for life expectancy in the Netherlands in the year 2000 [23]. In the first bid for each health state, respondents were offered half their remaining life expectancy in full health. Depending on the preference subsequently expressed, the time in full health was varied between half the life expectancy and 1/20 (if they preferred a short duration in full health) or 19/20 (if they preferred a longer duration in the offered health state) of the life expectancy. A ping-pong procedure was used until indifference emerged. The resulting score was expressed as proportion of the respondent's life expectancy.

- Time trade-off with 10 years (TTO-10) for the 6 health states: in this trade-off task, the period of time offered in the presented health state was fixed at ten years. The 10-year range in TTO-10 was deliberately chosen to be shorter than the actuarial life expectancies of all participants. The TTO-10 was similar to the TTO-LE in all other respects.

Other information collected in the questionnaire included biographical information – date of birth, sex, educational level (7 categories that were later combined into low (no secondary education), high (university degree or higher) and medium (all in-between)), whether they had children, religion, ethnicity, presence of illness in family or close friends, and information on the respondent's own health: presence or absence of 5 pre-specified medical condition(s) (listed as asthma or COPD, kidney disease, diabetes, arthritis, depression, and 'any other serious condition – please specify') now or in the past 5 years, respondent's own health state classified on the EQ5D+ and two ratings of one's own health, a verbal one (how would you rate your health in general – excellent, very good, good fair, or poor) and one using a VAS.

The session closed with evaluation questions about on the valuation tasks and the computerized questionnaire.

The feasibility of the questionnaire was extensively tested in a pilot study. A direct comparison between paper and internet questionnaire provided similar results.

### Data analysis

The educational level of our participants was compared with that in the Netherlands [24].

All VAS and TTO data were converted to scores ranging from 0 to 1. Respondents were removed from the analyses if they gave all health states the same valuations, or if they used the endpoints of a scale only.

Logical consistency in the valuation scores was analyzed as a quality check. Among the six health states, seven logical orderings could be defined. A health state description is logically better than another one if the attribute levels for all attributes are better or the same (e.g., 112121 is logically better than 222223). A logically better state is expected to be assigned a valuation score that is equal to or higher than the logically worse state; if not, this is called a violation of logical consistency. The distance between two logically ordered states is determined by the sum of the absolute differences between the attribute levels (e.g, the distance between 112121 and 222223 equals 1 + 1 + 0 + 1 + 0 +2 = 5). It is expected that logical consistency violations occur more often if the distance is smaller.

We calculated mean valuation scores, standard deviations (SDs), ranges, medians, and interquartile ranges for each health state and each method. VAS values differed by steps of 0.01 and TTO values by steps of 0.05 (1/20). Both TTO and VAS valuations were treated as continuous variables.

One-way analysis of variance (ANOVA) was used to test possible differences between the 3 age groups in the valuation scores for each of the six health states for each method (18 separate ANOVAs, dependent variable: health state valuation score, independent variables: 3 age groups as dummy variables).

Generalizability Theory (G-theory) was used as a general approach to estimate the relative contribution of the multiple sources to the total variance in the valuation scores [25]. G-theory is a specific application of ANOVA, deals with n-way designs and provides a flexible and practical framework to examine different sources of measurement error. G-theory extends classic test theory by recognizing and estimating the magnitudes of the multiple sources (facets in G-theory language) of variance. G-theory can be implemented within the ANOVA framework. In the present study, we used a generalizability study (G-study) to estimate, in a single analysis, the relative contribution of the independent variables or 'facets' (health states, valuations methods and respondents, see below) to the total variance in the valuation scores. The G-study allowed to attribute proportions of the total variance in the valuation scores to the health states valued, the elicitation methods (VAS, TTO-LE or TTO-10), and characteristics of the persons doing the valuing, using all data points simultaneously. We performed a G-study on all valuation scores from all methods collectively, and included 'health state valued', 'valuation method', 'age group' and 'respondent' as independent variables ('facets'). 'Respondent' includes all identified and unidentified respondent characteristics, and was nested under 'age group' because the two variables are dependent. We also included the first-order interaction terms. The second-order interaction was (by definition) included in the error variance component. If 'age group' had an independent effect on valuation scores, then 'age group' alone would explain a substantial amount of variance in the valuation scores. If an age effect was dependent on 'method', then the interaction term of 'age group' and 'method' would explain a large proportion of variance. If both 'age group' on its own and the interaction term with 'method' explained a large proportion of variance, both explanations might have held.

However, because a large proportion of variance appeared to be attributable to 'respondent' (see Results section), we analyzed which respondent characteristics were importantly correlated with health state valuation scores. First, we inspected measured characteristics (sex, educational level, having children, and health-related variables) for their correlation with the outcome variables (valuation scores) using Pearson correlations, one-way ANOVA, or Χ^2 ^tests, depending on the nature of the variable.

A p-value =< 0.01 was considered significant.

Subsequently, we used principal component analysis (PCA) and Ordination Based Cluster Analysis (ORBACLAN) to examine the data for response patterns [26,27]. ORBACLAN (available from the authors on request) is a divisive cluster analysis that follows the general principle of TWINSPAN [28], but uses Principal Components instead of correspondence analysis. The main problem with divisive cluster analysis is to reduce the number of possible dichotomies, which becomes too large to evaluate when the number of cases becomes relatively small (< 10). To find a suitable first division of the dataset a PCA is performed. The first axis can be regarded as the best way to order respondents on a continuous scale with a minimal loss of information. If we only test dichotomies by cut-off levels on the first ordination axis, we can be sure that this dichotomy will reflect the main variation in the dataset as reflected by the first PCA axis, but at the same time the possible number of dichotomies is reduced to N-1. This allows to evaluate all these dichotomies, and we choose the one that minimizes the pooled residual sum of squares in the two new clusters. For the largest of the two new clusters a new PCA axis is derived and the splitting procedure is repeated. The process stops when a predefined number of clusters is reached or the largest cluster (as measured by its residual sum of squares) is smaller than a predefined fraction of the sum of squares of the total dataset.

We predefined the number of clusters as 9, based on the number of respondents and the arbitrary limit of on average about 20 respondents per group. The mean score difference per health state between the scores in the clusters and the whole sample was calculated, which gives the direction of the deviation in the clusters.

Analyses were performed using the SPSS for Windows package, version 10.0 [29], except for the G-studies that were performed with the SAS package [30], and the cluster analysis that was performed with ORBACLAN [27].

## Results

### Respondents

Of the 606 subjects approached, 212 (100 males, 112 females) participated. Surveys were incomplete for 10 respondents. After excluding respondents who had not completed all valuation questions or whose data did not pass the quality checks, 187 complete surveys remained for analysis (Table 2).

**Table 2:** Mean respondent age (range) by age group and gender

	male		female		total sample	
	n	mean age (range*)	n	mean age (range*)	n	mean age
Age group 1	27	22.9 (19–26)	36	22.0 (17–28)	63	22.4
Age group 2	33	46.3 (39–50)	39	46.8 (41–53)	72	46.6
Age group 3	27	63.3 (60–72)	25	61.6 (55–65)	52	62.5

* ages of some respondents were outside the limits of the age groups approximately aimed at in the recruitment phase (see Methods)

Analysis of logical consistency showed on average 9.4% violations on the VAS (range 9% – 13% for the 7 pairs), 16.1% for TTO-LE (range 10%–29%), and 13.6% for TTO-10 (range 6% – 28%). The highest percentages of violations were found for 2 pairs with a distance of 2. There were no significant differences in the proportions of violations between the respondents who completed the questionnaire at the university or at home.

A total of 17% of respondents reported a low educational level, 48% a medium educational level, and 35% a high educational level (1% educational level unknown). Overall, the educational level of our respondents was significantly higher than the average national educational level (28%, 49% and 22% for low, medium and high educational level, respectively). A total of 55% of respondents had children, 35% was religious, 94% were of Dutch ethnicity, and 58% had experienced one or more medical conditions during the past 5 years. A total of 39% had not been involved with serious illness among family or close friends; 37% had but without care taking tasks, and 24% had care taking tasks for an ill person at least several times a week. Of all respondents, 77% judged their own health to be 'good' or 'very good'. The mean (range, SD) number of attributes affected (score > 1) on the EQ-5D+ classification was 0.94 (0–5, 1.21). The mean (range, SD) utility score on the EQ-5D [31] of the respondent's own health was 0.84 (-0.18 – 1.00, 0.23), and the mean (range, SD) VAS rating of own health was 83.5 (8–100, 14.0).

### Valuation scores for health states

Table 3 shows that the TTO scores had larger SDs and interquartile ranges than the VAS. Mean TTO-LE valuations were generally lower than TTO-10 and VAS valuations, suggesting an effect of time preference. TTO-10 confronted all respondents with a shorter life expectancy than their actual life expectancy, and this explains why the TTO-LE scores were generally lower than the TTO-10 scores.

**Table 3:** Mean scores (range), standard deviation, median and interquartile range of valuations of health states for VAS and two TTO variants, ranked from highest to lowest valuation.

	Mean (range)	SD	Median	Interquartile range
**VAS**				
112121	0.78^1 ^(0.20 – 1.00)	0.13	0.80	0.75 – 0.85
222111	0.70^1,2 ^(0.25 – 0.98)	0.15	0.70	0.65 – 0.80
122122	0.59 (0.10 – 1.00)	0.17	0.60	0.50 – 0.70
123121	0.57 (0.20 – 1.00)	0.15	0.60	0.50 – 0.65
332221	0.41^1 ^(0.00 – 1.00)	0.19	0.40	0.30 – 0.50
222223	0.38^1,2 ^(0.00 – 1.00)	0.22	0.35	0.25 – 0.50
				
**TTO-LE**				
112121	0.70^1,3 ^(0.03 – 0.98)	0.25	0.78	0.53 – 0.93
222111	0.68^1,3 ^(0.03 – 0.98)	0.25	0.73	0.53 – 0.88
122122	0.53^3 ^(0.03 – 0.98)	0.29	0.53	0.28 – 0.78
123121	0.49^3 ^(0.03 – 0.98)	0.30	0.53	0.23 – 0.73
332221	0.32^1,3 ^(0.03 – 0.98)	0.29	0.28	0.25 – 0.48
222223	0.24^1 ^(0.03 – 0.98)	0.28	0.08	0.25 – 0.43
				
**TTO-10**				
112121	0.80^3 ^(0.08 – 0.98)	0.18	0.88	0.68 – 0.98
222111	0.76^2,3 ^(0.03 – 0.98)	0.20	0.78	0.68 – 0.93
122122	0.60^3 ^(0.03 – 0.98)	0.27	0.63	0.48 – 0.88
123121	0.58^3 ^(0.03 – 0.98)	0.27	0.58	0.48 – 0.78
332221	0.38^3 ^(0.03 – 0.98)	0.30	0.38	0.03 – 0.63
222223	0.23^2 ^(0.03 – 0.98)	0.26	0.08	0.03 – 0.48

^1^significant difference VAS-TTO-LE

^2^significant difference VAS – TTO-10

^3^significant difference TTO-LE – TTO-10

### Correlation of age with the health state valuations

**Figure 1:**
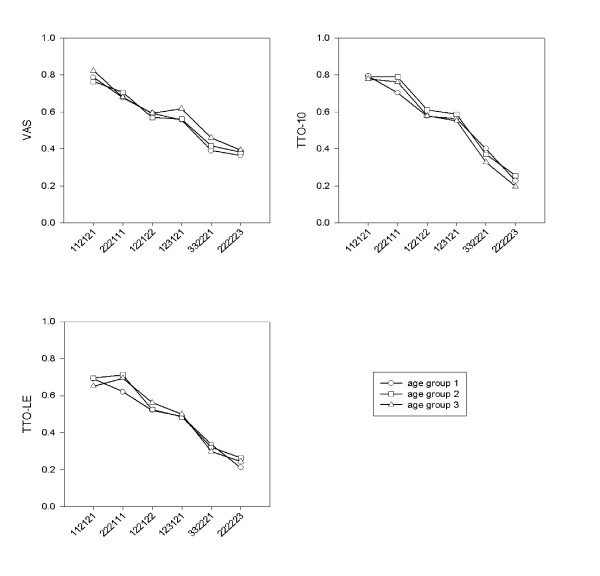
Mean valuation scores per age group (age group 1, mean age 22 years; age group 2, mean age 47 years; age group 3, mean age 63 years) per elicitation method: visual analogue scale (VAS), time trade-off with 10 years (TTO-10) and time trade-off with life expectancy (TTO-LE).

Average valuation scores per elicitation method are shown in Figure 1. Oneway ANOVA per health state per method showed no significant differences in valuation scores between the different age groups.

### Relative contribution of age and other characteristics to variance in valuation scores

Table 4 shows the results of a G-study including all valuation scores as dependent variables, and with 'health state', 'method', 'age' group, 'respondent' (nested under age), and interaction terms as independent variables (facets). 'Health state' explained the largest proportion of variance, followed by 'respondent' and the interactions of 'method' and 'health state' with 'respondent'. Thus, the variance in health state valuation scores was mainly attributable to differences in the descriptions of the health state, and secondarily by individual respondent characteristics (separately and in interaction with the health states and the elicitation method, respectively). Only a small proportion of variance was explained by the facet 'method' alone (1.6%), and only 0.2% of variance was attributable to age group.

**Table 4:** G-study on all elicitation methods collectively, incorporating the facets 'health state' (6 levels), 'method' (3 levels), 'age group' (3 levels) and 'respondent' (nested under age; 187 levels) and their interaction terms

*Facet*	% variance explained
*Main effects*	
Health state	36.8
Method	1.6
Age group	0.2
Respondent [age group]	11.1
*First order interactions*	
Health state * Method	1.4
Health state * Age group	0.0
Health state * Respondent [age group]	8.9
Method * Age group	0.0
Method * Respondent [age group]	17.3
	
Error	23.2

[age group] means 'nested under age group'.

Because we could not identify any relevant correlation between age group and the valuation scores, but identified an important effect of 'respondent', a subsequent analysis was conducted to identify respondent characteristics that were importantly correlated with the health state valuations. ANOVA showed no significant differences in valuation scores by sex, having children, being religious, judgment of own health state, or the reported presence of medical conditions now or during the past 5 years.

### Contribution of unmeasured characteristics: individual response patterns

Because measured respondent characteristics could not sufficiently explain the substantial contribution of 'respondent' to total variance in the valuation scores, we further investigated the nature of the response patterns of respondents. PCA revealed a first axis that explained 42% of variance; this means that there is a strong pattern in the responses. This axis was determined by a general tendency to give either high or low valuations on the TTO-10 and TTO-LE. The VAS scores were uncorrelated to this axis. Thus, this first axis indicates that 42% of total variance can be explained by respondents who use either the higher part or the lower part of the scale in TTO tasks, in combination with any VAS scores. The rank order of the health states was consistent across the methods, but explained less of the variation than the tendency to use high or low responses to the TTO tasks. The second, third and fourth axes, explaining 10%, 9% and 8% of variance, respectively, represented other systematic variations in the dataset, such as a high or low scoring tendency on the VAS, in various combinations with either high or low scores on specific health states on TTO-10 or TTO-LE.

Additional cluster analysis revealed nine clusters of response patterns in the data. Table 5 shows the direction and magnitude of the mean scores in the clusters, compared with the mean scores in the total sample. For instance, cluster 1 contained respondents with lower than average scores on all valuation methods, but especially on the TTOs. ANOVA did not reveal significant relations between the clusters and age or any other measured respondent characteristic (data not shown).

**Table 5:** Deviation, and direction of deviation, from the mean valuation scores in clusters found with Cluster Analysis (differences ≥ 0.20 marked in bold)

	Cluster (n)								
	1 (27)	2 (22)	3 (11)	4 (23)	5 (27)	6 (20)	7 (22)	8 (19)	9 (16)
VAS									
112121	-0.06	0.01	-0.01	0.02	0.00	0.01	-0.01	0.03	0.01
222111	-0.10	0.01	0.07	0.03	0.02	0.00	0.01	0.05	-0.06
122122	-0.16	-0.01	0.17	-0.01	-0.02	0.03	-0.05	0.15	0.02
123121	-0.05	-0.03	0.14	0.07	-0.07	-0.02	-0.04	0.12	-0.03
332221	-0.06	-0.09	**0.24**	0.10	-0.05	-0.08	-0.01	0.12	-0.03
222223	-0.14	-0.03	**0.32**	-0.08	-0.09	0.02	0.00	**0.25**	-0.04
									
TTO-LE.									
112121	**-0.40**	**0.25**	0.08	0.14	0.11	**0.22**	0.06	-0.13	**-0.31**
222111	**-0.29**	**0.24**	0.03	0.18	0.15	0.20	-0.07	-0.18	**-0.29**
122122	**-0.40**	**0.40**	0.16	0.13	0.02	**0.25**	0.03	**-0.34**	-0.17
123121	**-0.43**	**0.40**	-0.03	**0.31**	-0.19	**0.24**	0.11	-0.16	-0.18
332221	**-0.30**	**0.46**	0.15	**0.35**	**-0.24**	-0.01	0.07	**-0.26**	-0.14
222223	**-0.25**	**0.50**	**0.50**	-0.13	-0.14	0.04	0.05	-0.16	-0.18
									
TTO-10									
112121	**-0.28**	0.12	0.14	0.11	-0.02	0.11	0.04	-0.07	-0.03
222111	**-0.29**	0.16	0.09	0.15	-0.04	0.10	-0.01	-0.06	-0.03
122122	**-0.43**	**0.29**	**0.30**	0.02	-0.11	0.17	0.06	-0.15	0.11
123121	**-0.43**	**0.27**	0.16	**0.25**	-0.13	0.00	0.06	-0.12	0.11
332221	**-0.36**	**0.40**	0.14	**0.36**	**-0.31**	-0.15	**0.21**	-**0.20**	0.08
222223	**-0.24**	**0.46**	**0.46**	-0.15	**-0.23**	-0.03	0.15	-0.13	-0.00

## Discussion

The data reported in this paper were collected in a study aiming at disentangling two explanations for an effect of respondent age on TTO valuations of health states: a 'real' effect of respondent age on health state valuations and an 'artificial' age effect induced by the nature of the TTO task itself because of differential time preferences across age groups. We found no evidence for any effect of respondent age on valuations of health states. In a subsequent analysis, we identified individual response patterns that appeared to be unrelated to obvious, measured respondent characteristics.

Considerable differences in response patterns were found, probably reflecting individual differences in the cognitive use of the valuation scales inherent to the VAS and TTO tasks. Similar studies did not investigate response patterns, so we cannot judge whether our findings are exceptional. However, because it is not uncommon for TTO data to show large SDs (see, for instance, [32,33]), we suspect that response patterns also play a role in other studies. Krabbe et al. [32] found a larger proportion of variance explained by 'health state' (65% for the TTO method) and 10% of variance explained by 'respondent', but this was in a more homogenous respondent group (students). A second explanation is found in the selection of health states that were valued. In our study, the selection of states led to a smaller range in scores than in the study of Krabbe et al. Badia and coworkers [34], using face-to-face interviews in a random population sample, found a percentage of variance explained of 75% by 'health state', and 12% by 'respondent'. The proportion of variance explained by health states may be increased by selective sampling of respondents and/or health states, the use of direct questions allowing respondents 'correct' their response if it was 'incorrect' or illogical', and removing 'inconsistent' respondents from the analysis by applying using more strict criteria than ours; however, this remains a matter of speculation.

Different valuation scores were found for VAS and the two TTO variants, with TTO-LE resulting in lower valuations than TTO-10 and VAS. This suggests an effect of time preference. However, differences in valuation among respondents within a method were larger than differences among methods within a respondent (data not shown). A certain amount of variability among respondents within a method is to be expected, because of real differences in preference structure among respondents, and classical measurement error.

All respondents were presented with the health states and the valuation methods in the same sequence. As explained in the Methods section, there was a valid reason to present the VAS before the TTOs. The order we used may have affected the resulting scores due to anchoring or framing effects; however, it is difficult to speculate about the potential direction and we assume that the effect is smaller than the effects of individual response patterns. That we did not randomize the order of presentation of the health states can be considered a limitation of our study, as can he relatively low participation rate. Regrettably, in the Netherlands response rates of about 30% are common in general population surveys, and the task for the respondents was relatively demanding. In the recruitment of our volunteers we did not aim for a truly representative population sample. Given the original aim of the study, we aimed at contrasting data from respondents of different ages, and therefore sampled from 3 pre-specified age groups. We believe that the principal findings of our study are not adversely affected by the participation rate. The variability among respondents was sufficient to determine associations with the outcomes; collecting representative data was not our aim.

Our study further illustrates that large sample sizes are required to demonstrate significant relationships between demographic characteristics and values of hypothetical health states. A retrospective analysis of the power of our study showed that any difference in VAS scores for a separate health state between two age groups > 0.06 (on a 0 to 1 scale) would have been significant. Had the sample been 4 times larger, we would have been able to detect differences in scores per health state of 0.03. For TTO-10, we were able to detect differences of 0.10 (0.05 with a 4 times larger sample); and for TTO-LE, 0.09 (0.045 with a 4 times larger sample). At first sight, these results indicate that the study was insufficiently powered for the detection of age effects on valuation scores, especially for the TTOs. However, this seemingly low power is due to the fact that the individual variation in scores within age groups was larger than between age groups. We subsequently showed that individual response patterns (unrelated to age or other identifiable respondent characteristics) were the main source of the 'noise' in the scores. We then analyzed the 'noise' in the secondary data analysis. The power of the cluster analysis for detection of relations with age was much larger than in the separate analyses, because in the cluster analysis all outcome data (valuation scores) were combined: noise was removed and structure was kept. We concluded that these individual response patterns were not systematically related to age or to any other respondent characteristic measured in our respondents.

## Conclusion

This study shows that conventional methods for establishing health state valuations may be sensitive to individual response patterns when employed in the general population. In our study, these response patterns could not be explained by demographic respondent characteristics such as sex, age or educational level. Further valuation research, for example employing qualitative methods [35], should focus on explaining individual response patterns as a possible key to understanding the determinants of TTO scores.

## Abbreviations

ANOVA Analysis of Variance

PCA Principal components Analysis

QALY Quality adjusted life-year

TTO Time trade-off

SD Standard deviation

SG Standard Gamble

VAS Visual Analogue Scale

## Competing interests

The author(s) declare that they have no competing interests.

## Authors' contributions

MLE-B was responsible for writing the research proposal and the grant application. MLE-B, MCS, WJM and GJB developed the questionnaire. MCS was responsible for recruitment of the participants and data collection. MCS and CWNL analyzed the data, supervised by MLE-B, WJM and GJB.

MLE-B took the lead in writing the present manuscript. The writing committee consisted of MLE-B, MCS, WJM, CWNL and GJB. All authors contributed their comments on several versions of the manuscript and approved the final version that was submitted. The members of the VOTE group provided additional comments at relevant points in the course of the research project. MLE-B is the guarantor.

## Pre-publication history

The pre-publication history for this paper can be accessed here:


